# Descriptive epidemiological study of food intake among Japanese adults: analyses by age, time and birth cohort model

**DOI:** 10.1186/1471-2458-14-328

**Published:** 2014-04-08

**Authors:** Rei Otsuka, Hiroshi Yatsuya, Koji Tamakoshi

**Affiliations:** 1 Section of Longitudinal Study of Aging, National Institute for Longevity Sciences (NILS-LSA), National Center for Geriatrics and Gerontology, 36-3 Gengo, Morioka-cho, Obu, Aichi, 474-8511, Japan; 2 Department of Public Health, Fujita Health University School of Medicine, Nagoya, Japan; 3 Department of Public Health and Health Systems, Nagoya University Graduate School of Medicine, Nagoya, Japan; 4 Department of Nursing, Nagoya University School of Health Sciences, Nagoya, Japan

**Keywords:** Age effect, Diet, Japan, National survey

## Abstract

**Background:**

Although food and nutrient consumption among the Japanese population, known to have one of the longest life expectancies in the world, has changed markedly after World War II, little is known about the influences age, time and birth cohort have had. The present study examined the effects age, time and birth cohort have had on intake of 14 food groups from 1989 to 2009, using published data from the National Health and Nutrition Survey in Japan.

**Methods:**

The survey included 575 adults (271 men and 304 women) in 1989, 8431 (3952 men and 4479 women) in 1999 and 5632 (2629 men and 3003 women) in 2009. The effects of age on energy-adjusted food intakes defined by gender and birth cohort (birth in 1930–1939, 1940–1949, 1950–1959, 1960–1969, 1970–1979) were estimated using the mean polish process.

**Results:**

Intakes of meat and confectionary increased whereas those of milk and dairy products, sugar, and fats and oils decreased from 1989 to 2009. Both men and women in the 1940’s birth cohort consumed more fruit, although differences in food intake by birth cohort were less discernible. Furthermore, meat, fats and oils, and wheat intake decreased while fruits, fish, beans and vegetables consumption increased with aging in both men and women.

**Conclusions:**

The present analysis suggests intakes of meat and confectionary have increased in Japan over the past 20 years regardless of age and generation. Also, younger individuals are less likely to consume fruits, fish, beans and vegetables regardless of the birth cohort and time period. Differences in food group consumption by birth cohorts born between 1930 and 1979 were not obvious. The first indication of these findings would be that in order to avoid ongoing increases in meat and confectionery intake, the public health strategy should target the whole Japanese population. Secondly, intervening with the diet of younger individuals, especially today, would be reasonable as it is unknown whether today’s younger individuals will adopt a healthier diet when they age as the other generations did.

## Background

The Japanese diet is often considered synonymous with a healthy diet [1]. Food consumption patterns and nutrient intakes among Japanese people, however, have changed markedly in the last 50 to 60 years. Namely, along with overall westernization of the lifestyle, Japanese people as a whole now consume less carbohydrates, and more fat and meat than ever before [2-4]. For example, the percentage of energy obtained from carbohydrates decreased from 80.7% in 1949 to 57.5% in 2000, according to the National Health and Nutrition Survey in Japan (NHNS-J) [5]. In contrast, the percentage of energy obtained from fat increased from 6.9% to 26.5% during the same time period.

It has been suggested that fat intake (% energy) has increased, particularly among adults less than 60 years of age but not among older people [6], indicating possible differences by age or birth cohort. On the other hand, cross-sectional and longitudinal studies in Western countries indicate a decline in food intake with aging [7,8]. Total energy intake is reported to decrease with age [9], along with diminished taste and smell [10,11]. It is not known, however, if there is an effect of aging on food intake among Japanese.

Along with a significant change in the socio-economic environment after World War II, the nutritional status of Japanese people improved dramatically [5]. Nutritional education was implemented through maternal and child health activities [12], and nutritionally adequate school meals were initiated nationwide and spread rapidly [13]. These interventions that were targeted to a specific birth cohort or gender may have caused differences in nutritional knowledge and actual intake among birth cohorts and genders.

To accurately evaluate whether food intake has changed with age, it is necessary to separate time and birth cohort effects [14]. A birth cohort effect is a specific kind of age/time interaction. In theory, in the presence of a birth cohort effect, the influence of age is not the same for all calendar years, and conversely the influence of calendar year is not the same for all ages [15]. We, therefore, examined possible effects of age on certain food intakes using age, time, and cohort model in NHNS-J data for 1989, 1999 and 2009.

## Methods

### Data source

The Ministry of Health, Labour and Welfare of Japan has been conducting annual cross-sectional NHNS-J since 1945. Details of NHNS-J including its survey methods, historical background, and purpose, have previously been described [2,3,16]. Briefly, the survey includes three major components: clinical and laboratory assessment, dietary assessment, and a questionnaire on dietary habits, such as skipping breakfast and other lifestyle habits. Subjects in 300 randomly selected districts in Japan were surveyed annually.

**Figure 1:**
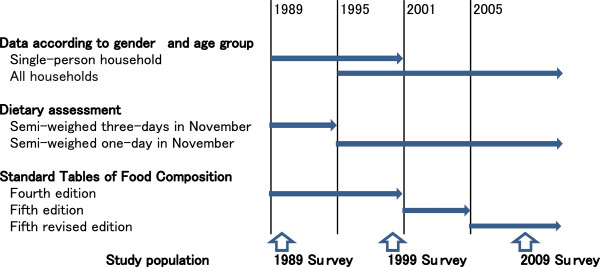
Publicly Available Data in National Health and Nutrition Survey in Japan from 1989.

Since individual-level data were not publicly available, we used publically available mean intakes of selected food groups according to age group in each survey year. In this study, we used published data from adults aged 20 or older who participated in the 1989 [17], 1999 [18] and 2009 [19] surveys (Figure 1). Food intake data according to gender and age group were not available before 1986. The 10-year interval between these three surveys was assigned in order to correspond with 10-year age categories used in the published NHNS-J results. Data were publicly available only for those 70 or older as one category, not 70–79, or 80–89. Therefore, we limited the analysis to 60–69 years in this study. The number of subjects, aged 20 to 69, involved in each survey was: 575 individuals (271 men and 304 women) in 1989; 8431 (3952 men and 4479 women) in 1999; and 5632 (2629 men and 3003 women) in 2009. Although the number of total study participants in the 1989 survey was comparable (approximately 20,000) to those in the 1999 survey (n = 15,000) and the 2009 survey (n = 15,000), gender- and age-stratified food and nutritional intakes were publically opened only for single-person households; therefore the number of subjects in the 1989 survey used in this study was relatively small.

### Dietary assessment

Semi-weighed three-day (1989 NHNS-J) and one-day (1999 and 2009 NHNS-J) dietary records were used to estimate the nutrient and food intake of individual participants. The recording was done in November, excluding Sundays and national holidays. In the semi-weighed method, all subjects were asked to weigh the amount of each food before and/or after preparation. If weighing the food was difficult (e.g. restaurant meals), estimated portion sizes were recorded [5]. When family members shared a dish, the household diet was recorded and used to estimate the nutrient and food intake for each family member according to the proportion of the shared dish that was consumed. Food intake in 1989 and 1999 was calculated based on the fourth edition, while 2009 food intake was based on the fifth revised edition of the Standard Tables of Food Composition in Japan [20,21]. In the 1989 survey, food intake at the single-person household level was published instead of mean food intakes *per capita*. Consequently, we used gender- and age-stratified food intakes for single-person households assuming that it represented individual intake.

In the present study, we selected 14 food groups from the Standard Tables of Food Composition for the analyses: 11 food groups (rice, wheat, potatoes, green yellow vegetables, other vegetables, fruits, milk and dairy products, meat, fish, beans, egg) with relatively high daily intake (>30 g/day), and three food groups (confectionery, fats and oils, sugar) that were considered important for understanding the secular and generational transition of the Japanese diet. Since intake from five food groups (nuts and seeds, mushrooms, algae, seasonings and spices, and prepared foods) were relatively low (<30 g/day), and beverage intake included both soft drinks and alcohol, we did not analyze these food groups. In order to avoid confusion between fat (nutrient) and fats and oils (food group), we quoted “fats and oils” when we refer to the food group.

We took into consideration changes in the coding system from the fourth to fifth revisions of the Standard Tables of Food Composition in the analysis. For example, rice was mainly coded as milled rice in 1989 and 1999 NHNS-J, but as steamed rice in 2009 NHNS-J [22]. The weight of steamed rice is about twice the amount of milled (raw) rice, e.g., when we steam 100 g of milled rice, the weight approximately doubles (212 g in the food composition table). We, therefore, divided rice intake in 2009 in half since steamed rice contains about the same quantity of water.

### Statistical analysis

To investigate the effects of age, time and birth cohort on food intake, the data were organized according to age group and time periods over the 10-year intervals, we created age-period contingency tables for each food intake, and simulated birth cohorts were constructed by combining age groups and time periods. The cohort effect is considered as a time (year)-age interaction since each cohort is uniquely defined by the pair of variables, age and year of the survey. Subjects aged 20–29 years in 1989, 1999 and 2009 were defined as the 1960–1969, 1970–1979 and 1980–1989 birth cohorts, respectively.

This investigation used a statistical model [23] symbolically represented by: *R*_*ij*_ = *a*_*i*_*+t*_*j*_*+c*(*k*) where *R*_*ij*_ (food intake) is modeled by the sum of the effects of the *i*th age group (i = 1 to 5, representing age groups from 20–29 to 60–69) represented by *a*_*i*_, the effects of the *j*th time period (1989 or 1999 or 2009 survey) represented by *t*_*j*_, and the effects of the *k*th cohort (birth at 1930–1939, 1940–1949, 1950–1959, 1960–1969, 1970–1979) represented by *c(k). c(k)* are the residual values, defined as the lack of additivity of age and time in determining *Rij*. We estimated the parameter *c(k)* by the mean polish process [23,24]. A *c(k)* value greater than zero indicates a greater than additive influence and less than zero indicates less than additive influence from age/time effects on food intakes. If no age/time interaction exists (no cohort influence), then the residual values will be small and close to zero. *C(k)* is defined as the residual of age/time interaction. It does not have reference groups. Instead, *c(k)* value of zero indicates no interaction, i.e. no cohort effect. Mean polish process does not provide information about statistical significance, but residual values obtained with this method provide a descriptive tool to detect and interpret interactions (lack of additivity) between two categorical variables, i.e. age and time [23].

In the analysis, food intakes (g/day) were adjusted for energy (g/ 4,186 kJ/day), using the energy density method, and expressed as food intakes (g/day)/energy intake (kJ /day) × 4,186.

## Results and discussion

The annual average food and energy intake are presented by gender age group in Table 1. Older age groups of both genders tended to eat more fruits, fish and less meat compared to their respective younger counterparts.

**Table 1:** Food and energy intake averaged over the years by gender and age group

		**Men**					**Women**				
	**Age group (years)**	**20-29**	**30-39**	**40-49**	**50-59**	**60-69**	**20-29**	**30-39**	**40-49**	**50-59**	**60-69**
Number of subjects (1989 Survey)^a^		129	46	36	35	25	36	23	41	82	122
Number of subjects (1999 Survey)		693	736	794	942	787	838	808	876	1011	946
Number of subjects (2009 Survey)		297	500	539	603	690	362	579	596	651	815
Energy intake	(kJ/day)	8941.2	9295.5	9497.7	9430.7	9140.6	7309.4	7695.8	7638.6	7875.7	7500.5
											
Rice^b^	(g/ 4,186 kJ/day)	206.2	205.5	211.1	215.8	207.7	136.4	146.6	147.8	157.0	154.4
Wheat	(g/ 4,186 kJ/day)	113.1	112.7	115.1	105.6	101.7	110.0	108.2	97.0	93.2	83.0
Potatoes	(g/ 4,186 kJ/day)	50.3	49.6	50.3	52.8	60.0	47.8	50.2	53.9	62.6	65.7
Green yellow vegetables	(g/ 4,186 kJ/day)	69.7	86.3	77.8	91.0	101.9	72.0	87.0	93.3	104.7	113.2
Other vegetables	(g/ 4,186 kJ/day)	152.9	162.8	170.0	190.5	206.0	143.4	159.9	174.6	183.9	183.6
Fruits	(g/ 4,186 kJ/day)	67.2	62.3	78.8	105.3	133.1	76.5	90.1	135.0	178.9	183.6
Milk, Dairy products	(g/ 4,186 kJ/day)	95.1	83.0	99.0	82.8	121.3	107.6	118.5	112.9	125.5	125.1
Meat	(g/ 4,186 kJ/day)	109.9	106.5	94.7	84.9	63.4	80.4	80.1	73.2	60.4	51.7
Fish	(g/ 4,186 kJ/day)	70.4	84.8	100.1	112.9	117.5	62.4	65.7	84.8	94.3	94.7
Egg	(g/ 4,186 kJ/day)	41.5	42.6	42.1	43.0	42.1	40.0	40.3	38.6	35.9	33.9
Beans	(g/ 4,186 kJ/day)	49.6	56.7	63.8	85.5	85.2	46.7	55.1	58.9	73.9	78.9
Confectionery	(g/ 4,186 kJ/day)	15.2	12.4	14.9	10.8	17.0	29.9	22.6	25.1	26.4	25.7
Sugar	(g/ 4,186 kJ/day)	8.0	9.6	9.2	9.2	9.4	7.7	8.3	9.6	10.6	10.2
Fats and Oils	(g/ 4,186 kJ/day)	19.6	19.7	17.9	16.7	12.7	17.2	16.7	16.1	15.1	11.7

Average energy, food and nutrient intakes were calculated from National Health and Nutrition Survey in Japan, 1989, 1999 and 2009.

^a^Available data for single-person household.

^b^Rice intake presented for 2009 was calculated as the half of that reported in 2009 NHNS-J.

### Differences by birth cohort (Birth cohort effect)

Differences in food intakes by birth cohort, often referred to as birth cohort effect are grouped according to gender and shown in Table 2. Positive values indicate more consumption in the particular birth cohort theoretically in any age group or at any time. Fruit intake in men was higher in earlier birth cohorts than in recent birth cohorts. On the other hand, fruit intake in women born in 1940–1959 was higher than that in birth cohorts born in 1930–39 or 1960–1979. We did not observe any differences by birth cohort for other food intakes examined in either gender.

**Table 2:** **Differences in food intakes by gender and birth cohorts**^**a **^**as estimated using the mean polish process**^**b**^

		**Men**					**Women**				
	**Year of birth**	**1930-39**	**1940-49**	**1950-59**	**1960-69**	**1970-79**	**1930-39**	**1940-49**	**1950-59**	**1960-69**	**1970-79**
Rice^c^	(g/ 4,186 kJ/day)	3.1	-3.6	-3.4	1.2	2.2	0.7	1.6	-1.4	-1.6	-0.2
Wheat	(g/ 4,186 kJ/day)	0.3	1.2	-0.2	0.0	-2.1	-0.6	-1.7	-0.4	0.1	2.6
Potatoes	(g/ 4,186 kJ/day)	1.0	0.1	-0.1	-0.5	1.4	1.5	0.0	-2.0	-0.7	0.5
Green yellow vegetables	(g/ 4,186 kJ/day)	-0.1	0.8	0.9	0.1	-0.7	-3.4	1.5	3.8	1.3	-2.5
Other vegetables	(g/ 4,186 kJ/day)	-1.3	-1.9	-0.2	-1.9	4.2	-2.7	-0.6	4.5	0.8	-5.4
Fruits	(g/ 4,186 kJ/day)	3.0	3.2	1.4	-2.3	-2.7	-5.2	5.4	7.6	-4.5	-5.3
Milk, Dairy products	(g/ 4,186 kJ/day)	-4.7	3.6	-4.1	-7.6	5.3	3.4	3.2	0.1	-2.8	-0.8
Meat	(g/ 4,186 kJ/day)	0.3	-1.9	0.3	1.2	0.8	0.5	-2.1	-1.4	-0.1	1.8
Fish	(g/ 4,186 kJ/day)	-3.3	4.2	0.0	-2.3	1.3	-1.8	-0.1	5.7	-0.4	-4.7
Egg	(g/ 4,186 kJ/day)	-1.0	-1.2	1.8	-0.5	-0.8	-0.4	-1.0	-0.3	1.0	0.1
Beans	(g/ 4,186 kJ/day)	3.4	-1.3	-1.7	-1.1	1.2	1.5	0.3	-0.9	-0.1	-0.1
Confectionery	(g/ 4,186 kJ/day)	-0.8	0.2	-0.2	-0.3	-0.5	1.8	-0.8	-1.2	-0.7	1.5
Sugar	(g/ 4,186 kJ/day)	0.2	0.3	0.3	-0.1	-0.3	-0.3	0.2	0.1	-0.4	0.0
Fats and Oils	(g/ 4,186 kJ/day)	-0.1	0.0	0.5	0.4	-0.6	-0.2	0.2	0.1	0.2	-0.2

Difference by birth cohort in food intakes are shown according to gender using the National Health and Nutrition Survey in Japan, 1989, 1999 and 2009.

^a^Minus symbol "-" means negative values. Positive values indicate more consumption in the particular birth cohort theoretically in any age group or at any time.

^b^A statistical model symbolically represented by: Rij = ai + tj + c(k) [23] where Rij (food intake) is modeled by the sum of the effects of the ith age group (i = 1 to 5, representing age groups from 20-29 to 60-69) represented by ai, the effects of the jth time period (1989 or 1999 or 2009 survey) represented by tj, and the effects of the kth cohort (birth at 1930-1939, 1940-1949, 1950-1959, 1960-1969, 1970-1979) represented by c(k). A c(k) value greater than zero indicates higher than additive influence and less than zero indicates lower than additive influence from age/time effects on food intake.

^c^Rice intake presented for 2009 was calculated as the half of that reported in 2009 NHNS-J.

### Differences by time (Time effect)

Differences in food intake by time period according to gender are shown in Table 3. Positive values indicate greater consumption in the particular period compared to 1989 in all age groups. Although caution is needed since there are differences in the survey methods used each year, consumption of meat and confectionery in both men and women, and of green yellow vegetables but not other vegetables in women, tended to be higher in the most recent survey. Similarly, less milk and dairy products, sugar, fats and oils were consumed by both men and women in the recent survey.

**Table 3:** **Differences by time**^**a **^**in food intakes according to gender estimated using mean polish process**^**b**^

		**Men**			**Women**			
**Time (year)**		**1989**	**1999**	**2009**	**1989**	**1999**	**2009**	
Rice^c^	(g/ 4,186 kJ/day)	0.0	-4.7	2.1	0.0	-9.9	-7.2	
Wheat	(g/ 4,186 kJ/day)	0.0	-9.9	0.6	0.0	-1.1	9.5	
Potatoes	(g/ 4,186 kJ/day)	0.0	12.7	9.1	0.0	8.0	1.2	
Green yellow vegetables	(g/ 4,186 kJ/day)	0.0	15.0	12.4	0.0	3.3	6.4	
Other vegetables	(g/ 4,186 kJ/day)	0.0	19.7	13.8	0.0	-1.3	-8.3	
Fruits	(g/ 4,186 kJ/day)	0.0	4.7	0.0	0.0	-15.5	-20.8	
Milk, Dairy products	(g/ 4,186 kJ/day)	0.0	-10.5	-16.2	0.0	-1.3	-8.3	
Meat	(g/ 4,186 kJ/day)	0.0	9.4	17.6	0.0	7.4	14.0	
Fish	(g/ 4,186 kJ/day)	0.0	8.2	-3.7	0.0	5.1	-5.9	
Egg	(g/ 4,186 kJ/day)	0.0	0.9	-1.7	0.0	-1.6	-2.9	
Beans	(g/ 4,186 kJ/day)	0.0	2.5	-6.2	0.0	4.3	-2.5	
Confectionery	(g/ 4,186 kJ/day)	0.0	0.9	2.2	0.0	1.8	3.2	
Sugar	(g/ 4,186 kJ/day)	0.0	-0.3	-1.8	0.0	-1.1	-2.5	
Fats and Oils	(g/ 4,186 kJ/day)	0.0	-1.2	-3.6	0.0	-0.7	-3.6	

Difference by time in food intakes according to gender are shown using National Health and Nutrition Survey in Japan, 1989, 1999 and 2009.

^a^Minus symbol "-" means negative values. Positive values indicate greater consumption in the particular period compared to 1989 in all age groups.

^b^A statistical model symbolically represented by: Rij = ai + tj + c(k) [23] where Rij (food intake) is modeled by the sum of the effects of the ith age group (i = 1 to 5, representing age groups from 20-29 to 60-69) represented by ai, the effects of the jth time period (1989 or 1999 or 2009 survey) represented by tj, and the effects of the kth cohort (birth at 1930-1939, 1940-1949, 1950-1959, 1960-1969, 1970-1979) represented by c(k). A c(k) value greater than zero indicates higher than additive influence and less than zero indicates lower than additive influence from age/time effects on food intake.

^c^ Rice intake presented for 2009 was calculated as the half of that reported in 2009 NHNS-J.

### Differences by age (Age effect)

Differences in food intake by age according to gender are shown in Table 4. Positive values indicate greater consumption in the particular age group compared to theoretical values for the 20-29-year age group in any generation or at any time point. Meat intake decreased with an increase in age in both genders; the intakes in men and women in the 60–69 year age group were 22.2 g/4,186 kJ and 18.3 g/4,186 kJ lower than the 20–29 year age group in men and women, respectively. Intakes of fats and oils and wheat also decreased in older men and women. In contrast, intake of fruits, vegetables, fish, and beans increased in older age groups of both genders. Intake of rice and potatoes in men did not differ considerably according to age group while it increased with aging in women. Similarly, intake of confectionery and sugar were slightly higher in older age groups, but only in women. Egg intake remained constant, and no clear trends were observed in the intake of milk and dairy products by age group in either men or women.

**Table 4:** **Differences by age**^**a **^**in food intakes according to gender estimated using mean polish process**^**b**^

		**Men**					**Women**				
**Age group (years)**		**20-29**	**30-39**	**40-49**	**50-59**	**60-69**	**20-29**	**30-39**	**40-49**	**50-59**	**60-69**
Rice^c^	(g/ 4,186 kJ/day)	0.0	-4.0	-3.6	-0.9	-1.6	0.0	1.2	3.5	6.3	17.6
Wheat	(g/ 4,186 kJ/day)	0.0	-2.3	-2.2	-6.2	-6.4	0.0	-5.6	-9.3	-12.6	-14.8
Potatoes	(g/ 4,186 kJ/day)	0.0	-1.0	-1.1	0.1	4.1	0.0	2.0	5.8	9.1	11.8
Green yellow vegetables	(g/ 4,186 kJ/day)	0.0	6.5	2.0	8.1	14.3	0.0	3.8	8.6	16.1	16.3
Other vegetables	(g/ 4,186 kJ/day)	0.0	2.2	3.9	13.4	23.2	0.0	8.3	10.8	15.6	14.4
Fruits	(g/ 4,186 kJ/day)	0.0	-3.4	3.2	15.3	29.5	0.0	24.5	45.6	53.7	47.5
Milk, Dairy products	(g/ 4,186 kJ/day)	0.0	-6.9	-0.9	-7.6	11.2	0.0	-2.6	2.0	5.4	-3.3
Meat	(g/ 4,186 kJ/day)	0.0	-3.3	-9.3	-13.5	-22.2	0.0	-3.5	-11.6	-15.0	-18.3
Fish	(g/ 4,186 kJ/day)	0.0	5.4	11.2	17.2	21.0	0.0	10.5	14.5	17.3	16.6
Egg	(g/ 4,186 kJ/day)	0.0	-0.2	-0.8	-0.3	-0.1	0.0	-0.7	-2.7	-2.9	-0.6
Beans	(g/ 4,186 kJ/day)	0.0	2.4	5.0	14.8	15.9	0.0	2.3	9.3	14.0	13.7
Confectionery	(g/ 4,186 kJ/day)	0.0	-1.5	-0.5	-2.3	0.7	0.0	1.3	1.6	1.7	1.7
Sugar	(g/ 4,186 kJ/day)	0.0	0.5	0.3	0.3	0.5	0.0	0.7	1.1	1.2	1.6
Fats and Oils	(g/ 4,186 kJ/day)	0.0	-0.4	-1.4	-1.9	-3.5	0.0	-0.3	-1.1	-2.5	-3.3

Difference by age in food intakes according to gender are shown using National Health and Nutrition Survey in Japan, 1989, 1999 and 2009.

^a^Minus symbol "-" means negative values. Positive values indicate greater consumption in the particular age group compared to theoretical values for the 20-29-year age group in any generation or at any time point.

^b^A statistical model symbolically represented by: Rij = ai + tj + c(k) [23] where Rij (food intake) is modeled by the sum of the effects of the ith age group (i = 1 to 5, representing age groups from 20-29 to 60-69) represented by ai, the effects of the jth time period (1989 or 1999 or 2009 survey) represented by tj, and the effects of the kth cohort (birth at 1930-1939, 1940-1949, 1950-1959, 1960-1969, 1970-1979) represented by c(k). A c(k) value greater than zero indicates higher than additive influence and less than zero indicates lower than additive influence from age/time effects on food intake.

^c^Rice intake presented for 2009 was calculated as the half of that reported in 2009 NHNS-J.

## Discussion

In the present study, differences in food intake by age, time, and birth cohort were examined using 1989, 1999 and 2009 NHNS-J. The main findings of the present study are three-fold. First, intake of certain foods changed in a steady pattern with aging. Second, intake of certain foods also changed in a steady pattern with time between 1989 and 2009. Third, differences by birth cohort for food intake were less discernible.

Meat, fat, and oil intakes were lower in older age groups of both genders than the younger groups. This finding is consistent with previous longitudinal studies that found decreases in total fat consumption with aging among older Americans [25] or older Australian women [26]. A potential explanation for the lower fats and oils and meat intakes among older adults is the decline in digestive function and energy requirements that occur with aging in relation to reduced physical activity [27,28].

Intake of fruits, fish, beans, and vegetables were higher in older age groups of both genders than younger groups. Since intake of these foods is encouraged in the “Dietary Guidelines for Japanese” [28] or “Health Japan 21” [29] set forth by the Ministry of Health, Labour and Welfare of Japan [30] aimed at preventing lifestyle-related diseases, and these foods are rich in the traditional Japanese diet [31], older individuals may be more prudent in their choice of foods compared to younger individuals. This would be natural as older individuals are more aware of associations between diet and health, and they also may be more conscientious if they or their peers suffer from ill-health. In other countries, which are consistent with our findings, several longitudinal studies indicate that the elderly make positive changes to their diet. Prynne *et al.* reported that fat intake decreased, while intake of fruits and vegetables increased in 1253 men and women born in 1946 from the 17 year British birth cohort study [32]. Fernyhough *et al.* reported that meat intake decreased in men and women during their 6-year follow-up of a community dwelling in adults aged 70 years or over in New Zealand. They examined the association between age, time, and cohort effects, and they also reported that older adults, particularly women, were making healthier food choices. For example, women ate brown or whole-wheat bread at the 6-year follow-up [33].

It is also possible that food palatability changes with aging, thus increasing preference for these foods in older individuals [9]. The main sources of protein among Japanese in 2008 NHNS-J were fish (22%), meat (18%) and beans (7%) [34]. Assuming that total protein intake remains stable, higher intakes of fish and beans, and lower intakes of meat among older individuals assessed in the present study may indicate a shift from meat to fish and beans since they are easier to chew. The textures of tofu (bean curd), which is made from beans, and fish are softer than meat, and older individuals may prefer eating these softer foods due to decreased oral function.

Our finding that younger individuals consumed less fruits, fish, beans, and vegetables indicates that public health strategies likely need to pay more attention to these age groups in order to achieve the goal of the Japanese health promotion plan called “Health Japan 21” by the Ministry of Health, Labour and Welfare of Japan [30].

Differences in food intake by birth cohort were relatively small in the present study. Only fruit intake in men tended to be higher in earlier birth cohorts than in recent ones. For example, fruit intake of men born in 1930–1939 was 5.7 g/4,186 kJ/day higher than that in men born in 1970–79. Fruit consumption would be influenced by environmental and host factors, such as household income, marital status and food availability [35]. Indeed, compared with recent birth cohorts, higher rates of marriage [36] and average savings [37] are reported among earlier generations of Japanese men and these variables may influence the higher fruit intakes.

Some studies reported that differences exist in body mass index [38] or in mortality from specific cancers [39-41] according to birth cohort, and speculated that these differences could be attributed to differences in birth cohort diet. Our findings, however, do not corroborate these speculations. Since all subjects in the present study were born between 1930 and 1979, and the majority of them were born or educated after World War II, they might have had similar nutritional knowledge, making potential effects of birth cohort difficult to detect.

In the present analysis, we also found a tendency for increased intake of meat and confectionery in both men and women in recent surveys. In contrast, intake of milk and dairy products, sugar, and fats and oils decreased in recent surveys in both men and women. Although some of these observed changes may be due to variations in the survey methods over the years, they may indicate that the Japanese diet is shifting toward higher intakes of meat and confectionery, and lower intakes of milk and dairy products, sugar and fats and oils. Even though we found in the present study that intakes of “fats and oils” decreased in Japan in the last two decades, the percentage of energy obtained from fat increased from 6.9% to 26.5% between 1949 and 2000 NHNS-J [5]. However, in line with the present finding, within 1989 to 2009 NHNS-J data, the values fluctuated very little, i.e., the energy obtained from fat was 25.7%, 26.5% and 25.6% of total energy in 1989, 2000 and 2009, respectively [5,19]. Consumption of meat and animal food products in the last few decades also increased in South Korea, India, Spain, the Middle East and North Africa [42-45]. These studies, however, did not examine possible effects of time, age, and birth cohort effects have on diet.

Even though intake of milk and dairy products is encouraged in Japanese dietary guidelines, or “Health Japan 21”, since these products provide a good source of calcium, vitamins and protein, their consumption decreased over the past 20 years. Attention should, therefore, be paid to the time trends in milk and dairy intake in order to achieve the goals of “Health Japan 21”.

### Study strengths and limitations

Strengths of the present study include the examination of independent associations of age, time and birth cohort with certain food groups and energy intakes by separating out the other two effects with the use of publically available data on the general Japanese population born between 1930 and 1979. Also, the fact that the mean polish method does not require a special statistical package makes the present and present-like analysis easily reproducible. We believe public health programs for chronically emerging conditions should be designed after the elucidation of possible age, time and birth cohort effects.

One major limitation of this study is that only data from 1989, 1999 and 2009 NHNS-J were used in the analyses. Although NHNS-J has been conducted since 1945, gender- and age-stratified data were not available before 1986. Furthermore, we required 10-year intervals between three surveys in order to correspond with the 10-year age categories used in the published NHNS-J results. In addition, gender- and age-stratified nutritional intake *per capita* in 1989 used in the analysis was derived from NHNS-J participants living in a single-person household. The representation of single-person households may be limited. However, the difference in nutritional intake *per capita* between single-person and full households in 1989 was not large. For example, the mean *per capita* energy intake in each type of household was 1,980 kcal (8,288 kJ) and 2,061 kcal (8,627 kJ) per day, respectively [18]. Furthermore, we used half quantity (g/day) estimates for rice intake in 2009 to take into account the difference in the food code used in each of the surveys. Food intakes in 1989 and 1999 were calculated based on the fourth edition, while 2009 was calculated based on the fifth revised edition of the Standard Tables of Food Composition in Japan. The classification criteria of some foods into specific food groups changed with the revision; for example, fruit jam was classified into the sugar group in the fourth edition, but was classified into the fruit group in the fifth revised edition.

The time effect in this study, i.e., the time trend between the 1989 and 2009 surveys, might therefore have been influenced by changes in the coding practice that occurred with the revision. It is necessary, in future analyses, to calculate food intakes according to the same standard tables. With regard to age effects, men and women consumed more fruits, fish, beans and vegetables with aging, and less wheat, meat, and fats and oils. A longer-term study, in which the same subjects are measured repeatedly, would be needed to clarify the aging-related changes. Finally, the present study using the mean polish process should be interpreted in a descriptive manner, and further studies using statistical methods such as analysis of variance are needed to test the described findings.

## Conclusions

In summary, the present analysis suggests that meat and confectionary consumption have been increasing in Japan in the past 20 years regardless of the age and generation. Also, younger individuals are less likely to consume fruits, fish, beans and vegetables regardless of the birth cohort and time period. Differences in food group intakes by birth cohorts born between 1930 and 1979 were not obvious. These findings indicate firstly that, the whole Japanese population would need to be targeted in order to avoid ongoing increases in meat and confectionery intake. Secondly, prioritizing younger individuals of today for dietary intervention would be reasonable as it is unknown whether today’s younger individuals adopt a healthier diet when they age as the generations did before.

## Abbreviations

NHNS-J: National Health and Nutrition Survey in Japan.

## Competing interests

The authors declare that they have no competing interests.

## Authors’ contributions

The work was carried out in collaboration among all the authors. RO and HY defined the research theme, HY designed the methods, and RO analyzed the data, interpreted the results and drafted the manuscript. KT supervised the analyses, interpreted the findings, and contributed to critical revision of the manuscript. All authors read and approved the final manuscript.

## Pre-publication history

The pre-publication history for this paper can be accessed here:

http://www.biomedcentral.com/1471-2458/14/328/prepub
